# Exploratory Study on Occupational Health Hazards among Health Care Workers in the Philippines

**DOI:** 10.29024/aogh.2316

**Published:** 2018-08-31

**Authors:** Erwin Martinez Faller, Nataman bin Miskam, Adrian Pereira

**Affiliations:** 1 School of Pharmacy, Management and Science University, 40100, Shah Alam, Selangor, MY; 2 Migrant Workers Health Research Network, Shah Alam, Selangor, 40100, MY; 3 Global Health Pharmacy Network, Shah Alam, Selangor, 40100, MY; 4 International Consultant, Petrosphere Inc, Puerto Princesa, 5300 Palawan, PH; 5 Pejabat Kesihatan Daerah Klang, Ministry of Health, Klang, Selangor, MY; 6 North-South Initiatives, Petaling Jaya, MY

## Abstract

**Background and Purpose:**

Healthcare workers are prone to occupational hazards. The study aims to identify the occupational health hazards among healthcare workers in the Philippines and its essential relevant developmental framework. This article evolved on the responses of participants on how they can improve strategies and barriers for healthcare workers to comply with Occupational Health and Safety (OSH).

**Methods:**

A qualitative study design in which 15 healthcare workers from nurses (4), pharmacists (3), medical technologies (4) and medical doctors (4) participated: two focus group of three to four participants each and eight in-depth interviews. The thematic sessions were identified, including occupational health and safety policy implementations, hazards experiences, barriers, and strategies for quality improvement for OSH. Focus groups and interviews using transcript-based analysis were identified relating to emerging themes on the challenges they had experienced while accessing provisions of OSH in their workplace.

**Results:**

Majority of the participants revealed the existence of policy on Occupational Health and Safety (provisions, guidelines and regulations on OHS from the government) and mentioned that there were limited OHS officers to supervise the healthcare workers in their workplace. Some have limited accessibility to the requirements of the implementation of OHS (free facemasks, gloves, disinfectants, machines, OSH staff, etc.) among healthcare workers, while the workload of the staff in the implementation of OHS in the workplace gradually increased. The results indicated that the respondents were knowledgeable in the implementation of OHS in the workplace, and that there was no existing ASEAN framework on the protection and promotion of the rights of healthcare workers in their workplace. Facilities need to improve health assessment, and to ensure constant evaluation of the existing laws for healthcare workers (quality assurance of existing policies) in their working areas. Direct access to OSH officers, occupational hazards education, emergency contact etc. must be improved. Adherence must be strengthened to fully comply with the OHS standards.

**Conclusion:**

The researchers inferred that issues and concerns regarding compliance on provisions of occupational health and safety among health care workers must be properly addressed through immediate monitoring and reevaluation of personnel in terms of their knowledge and practices in OHS. Barriers and challenges have been identified in the study that can lead to improved compliance among healthcare workers in regards to OHS.

## Introduction

Occupational safety and health (OSH) is a multidisciplinary field concerned with the safety, health, and welfare of people at work. In doing so, the United States Centers for Disease Control and Prevention (US CDC) have placed standard precautions that aim to reduce the risk of transfer of disease-causing viruses or bacteria (generally called pathogens) from the blood or other moist regions of the body—such as mucous membranes and damaged skin—that can harbor them [1].

Provisions on occupational health and safety were quite relevant during the SARS outbreak in 2003. At the time, no known treatment for SARS was available, and strict adherence to infection control guidelines and policies and procedures were the only recognized effective method to prevent and control the global spread of this dreaded disease. Findings revealed substantial deficiencies in knowledge, practice and policy compliance among health care providers. The World Health Organization (WHO) and the Philippines Department of Health (DOH) realized the need to strengthen the nationwide Infection Control Program in order to enhance the preparedness of health care workers, so that they would be able to respond to outbreaks of highly transmissible infectious diseases and, more importantly, to prevent and reduce occurrence of health care-associated infections among patients [2,3].

Some provisions of Occupational Health and Safety call for the adoption of safe practices for handling needles and other sharp objects, in order to prevent further outbreaks, especially of Hepatitis B and C, which are frequently associated to healthcare providers. A breach in infection control practices facilitates the transmission of infection from patients to health care workers, and other patients and attendants. It is therefore important for all health care workers to adhere to the Strategies on Occupational Health and Safety. It is also imperative for health care administrators to ensure implementation of the OHS in their respective facilities [4].

National evidenced-based guidelines for preventing healthcare-associated infections in National Health Service (NHS) Hospitals in England emphasize the lack of, or insufficient knowledge of, occupational health and safety protocols as a major cause of a number of outbreaks in healthcare settings. The NHS in Scotland, for example, raised awareness of the importance of hand hygiene. Hand hygiene compliance across NHS boards in Scotland has been achieved by the use of a standardized approach involving an audit tool and improved protocols [5]. Likewise, barriers to the implementation of occupational health and safety provisions can be correlated to the age and gender of respondents as well as education, motivation or system [6]. It was shown that these variables can influence the adherence of healthcare workers [7].

In one study, female participants may have been more apprehensives than male about infection control in their workplace. This is because females may have a greater risk for urinary tract infections than males as the result of havingan anatomically shorter urethra [8].

According to the De Castro *et al*. (2009), nurses in the Philippines experience being injured on the job once or twice in the past year on average, and 6% have been injured at least three times. More than three-fourths (78%) experienced back pain and more than half (53%) continued working despite this pain [9].

For the most part, injuries and occupational health hazards are not well studied and recorded for communication with other health care workers. Because of this, the current study aims to identify the occupational health hazards of health care workers in the Philippines. The OHS procedures have been recognized as being an efficient means to prevent and control associated infections, especially in the hospital setting. Such measures not only protect health care workers, but also improve the working environment.

## Method

A qualitative study design in which 15 healthcare workers from nurses (4), pharmacists (3), medical technologies (4) and medical doctors (4) employed currently in Phillippine hospitals participated: two focus groups of three to four participants each and eight in-depth interviews. A formulated theme for the individualized interview and focus group discussion (FGD) was chosen based on the semi-structured format as main focal points: occupational health hazards as experienced in the workplace; implementations; and barriers and strategies for quality improvement on OSH in the workplace. The information recorded in this study was carried out to participants through phone calls, physical meetings and email messages. Convenience sampling design was used to access the source of data both in FGD and in-depth interview. Data collection took part from September to December 2017. Questionnaires for in-depth discussion and FGD were validated by experts. The inclusion and exclusion criteria on the selection of respondents were based on their reliability to discuss and provide input on the challenges facing among healthcare workers in the hospitals.

Two focus group discussions were conducted to provide perceptions and opinions on topics in the selected themes based on the following steps outlined in Krueger [10]. Both groups employed convenience sampling wherein mixed groups of participants from migrant worker’s leaders, NGO and health and safety officers attended. All potential FGD participants were given a formal invitation and contacted via mobile phone for setting up the schedule.

In addition, eight in-depth, semi-structured interviews were conducted withhealth professionals of different backgrounds. Each interview lasted approximately 30 minutes and was audio recorded. Transcript-based analysis was conducted following the techniques suggested by Krueger [10]. Ethics and confidentiality was assured during the study.

## Results and Discussions

**Figure 1:**
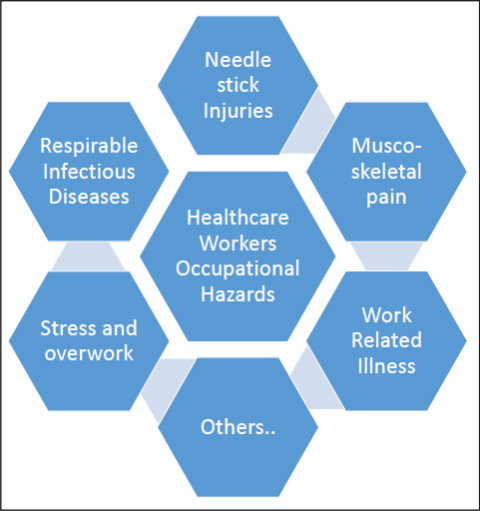
Healthcare Workers’ Identified Occupational Hazards.

The 15 respondents were chosen from various health care professionals (pharmacists, medical technologists, nurses and medical doctors). All of them were working in tertiary hospitals. It can be noted, however, that the interviewed healthcare professionals were often exposed to various occupational health hazards with required safety protocols as they can be exposed to bloodborne pathogens, sharp objects (e.g. syringe), cytotoxic agents, medical waste products, radiation, and other forms of health risks. Nursing was identified as a hazardous occupation compared to the other healthcare roles due to direct patent interactions [9]. They are unusually prone to enormous amounts of “workload and stress” that may lead to other health and safety concerns (Figure 1).

Results demonstrated that existence of current laws on occupational health and safety and the presence of OHS officers (within the organization) that supervise healthcare workers that may lead to compliance among them on occupational health and safety provisions, guidelines and regulations. Based from the results, one participant was quoted saying that “There were supervisors that routinely check us if we comply to the policy OHS”. Another participant said, “It is part of the training, prior to employment that supervisors ensure that the newly hired HCW [healthcare workers] have read and understand the safety procedures.” One major aspect of the interviews indicated that the “HCW safety manual is also necessary”. This established the need to reinforce the safety and security manual, provide training to new employees, and retraining of workers are all necessary to improve compliance and overcome challenges in complying with OHS (Table 1).

**Table 1:** Highlights of respondents talk about quotes from focus groups and in-depth individual interviews.


Occupational Hazards Experiences: “I sometimes poke myself with the needle, not only once but many times in a year” “I’m stressed about my work due to a lot of work” “I’m almost sick for more than 2 days due to my work”
Barriers and challenges observed: “There were supervisors that routinely check us to see if we comply to the OHS policy” “It is part of the training, prior to employment, that supervisors ensure that the newly-hired HCW have read and understand the safety procedures” “The actual and appropriate practices among the staff on OHS are strictly followed only when strong policy towards this practice is available to employees”
Suggestions on quality improvement: “HCW safety manual is also necessary” “Nonetheless compliance on the policies and guidelines of occupational health and safety lies on the personnel designated to do the job. Though each of us is obliged to adhere to the implementing rules and regulation towards infection control, but implementation of such guidelines is best shown when people designated to do the task (e.g. disposal of waste) are doing it routinely as part of their daily tasks”


**Figure 2:**
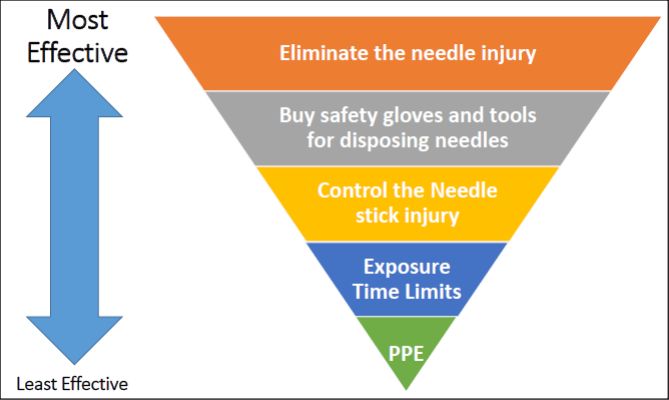
Strategies to avoid needle stick injury using Hierarchy of Control.

Increases in requirements is another barrier to compliance and implementation. On the course of the actual interview with the participants, one was quoted as saying that “The actual and appropriate practices among the staff on OHS are strictly followed only when strong policy towards this practice is available to employees”. In addition, one of the participants said that “Nonetheless compliance on the policies and guidelines of occupational health and safety lies on the personnel designated to do the job. Though each of us is obliged to adhere to the implementing rules and regulation towards infection control but implementation of such guidelines is best shown when people designated to do the task (e.g. disposal of waste) are doing routinely as part of their daily task”. Accordingly, strategies can be employed to develop a framework for improving the occupational safety and health quality in the workplace. A hierarchy of controls can be used as a means of determining how to implement feasible and effective control solutions to existing problems (e.g. needlestick injury hazards) as often used by safety organizations (Figure 2) that have the most effective known processes [11]. In addition, OSH can be achieved thru improvement in knowledge of proper hygiene and compliance that can ultimately improve a patient’s outcome [12].

## Limitations

Although the participants were health care workers from different regions in the Philippines, the respondents in this qualitative sample were known by the researchers in the hospital settings. Therefore, the readers should be mindful that findings of this study cannot be generalized to the entire archipelagic regions in the Philippines. Indicators used in the study do not capture the complexity of working descriptions (e.g. intensive care unit nurses, emergency room doctors, etc.) Future studies can employ quantitative methods to complement and extend the current study.

## Conclusions

Based on the findings of the study, it can be inferred that the issues and concerns discussed on occupational health and safety among health care workers during the interview calls for the immediate monitoring and evaluation of HCP policies and procedures. In this manner, compliance to the provisions, guidelines and protocols in occupational health and safety can be strictly followed.
